# Neonatal factors impacting umbilical cord blood unit characteristics

**DOI:** 10.1038/s41598-025-96829-3

**Published:** 2025-05-14

**Authors:** Ahmad Darwish, Mohamed Reda Bassiouny, Ahmed Kamal Mansour, Sohier Yahia, Sara Mohamed, Ashraf El-Ghazali, Nora El-Tantawy, Mohamad Darwish

**Affiliations:** 1 Pediatric Department, Faculty of Medicine, Mansoura University, Mansoura, Egypt; 2 Faculty of Medicine, Mansoura Research Center for Cord Stem Cells (MARC-CSC), Mansoura University, Mansoura, Egypt; 3 Obstetric and Gynecology Department, Faculty of Medicine, Mansoura University, Mansoura, Egypt; 4 Clinical pathology Department, Faculty of Medicine, Mansoura University, Mansoura, Egypt; 5 Medical Parasitology Department, Faculty of Medicine, Mansoura University, Mansoura, Egypt; 6 Public health Department, Faculty of Applied Medical Sciences, Al-Baha University, Al-Baha, Saudi Arabia

**Keywords:** UCB, Neonates, Bankability, TNC, CD34+, Stem cells, Stem cells, Medical research

## Abstract

A promising alternative to bone marrow in hematopoietic stem cell transplantation is umbilical cord blood (UCB). Major barrier to its use in transplantation is stem cell quantity and quality. It is crucial to determine the variables impacting the quality of these cells for bankability. The study aimed to investigate the impact of neonatal factors on UCB units. A total of 150 UCB units that were collected during the caesarean section were included in the study. The sex, birth order, gestational age, birth weight, chest circumference, head circumference, and Apgar score of the newborns were recorded after delivery. The cord blood volume was calculated. The numbers of CD34 + cells and total nucleated cells (TNCs) were determined. Univariate analysis revealed that larger babies, heavier placental weights, increased head and chest circumferences, and longer umbilical cords were associated with greater volumes of cord blood and higher CD34 + and TNC cell counts. A greater UCB volume and a higher CD34 + cell count was associated with a longer gestational duration. To determine the primary selection criteria and estimate the yield, a multivariate linear regression analysis was used. Heavier placentas had higher TNC and CD34 + cell counts and greater cord blood volumes. Larger babies gave UCB units with increased volume. Longer gestational-age newborns had a higher CD34 + cell count in their UCB unit. Our findings suggest that placental weight is the key predictive variable influencing the quantity and quality of UCB units, which is essential for successful cord blood transplantation and bankability.

## Introduction

Banking of umbilical cord blood (UCB) has increased in importance over the previous 30 years because of advances in cord blood transplantation and its application in regenerative medicine^1^. When hematopoietic stem cells are collected for transplantation, UCB is considered to be a better option than bone marrow and peripheral blood. Treatments for metabolic illnesses and hematological malignancies may involve the use of hematopoietic stem cells obtained from cord blood^2^. Compared with alternate hematopoietic stem cell sources, cord blood provides various advantages, including immediate availability, no risks to donors, convenience in harvesting, lower risk of graft-versus-host conditions, and less demand for HLA matching^3^.

Across 36 countries, approximately 51 recognized public cord blood banks have preserved more than 800,000 cord blood units^4^. The restricted quantity of stem cells that can be extracted from every sample is the primary constraint on the use of UCB in transplantation. One to three% of bone marrow cells, 0.1–4% of UCB cells, and 0.01–1% of peripheral blood cells are CD34 + positive^5^. The quality of the UCB units that are preserved determines the success of the transplant^6^. The volume, TNCs, and CD34 + cell count are crucial factors in determining eligibility for CB banking. After UCB transplantation, engraftment and survival have been linked to the quantity of CD34 + T cells^7,8^. Furthermore, the volume of UCB units is one of the most important factors for banking. The minimum volume recommended ranges from 40 to 60 millilitres^9^. A minimal criterion of ≥ 2 × 10^7^/kg total nucleated cells (TNCs) in cord blood has been recommended by various cord blood banks for UCB transplantation^10^.

One financial challenge for advancements in the field of public cord blood banking is the limited amount of space available for preserving cord blood units. Owing to the decreased cell count, prior to cryopreservation, more than half of the UCB units collected for banking are wasted^11,12^. To save costs and avoid keeping unneeded units of cord blood, it is crucial to select a donor who will provide the best-quality cord blood units appropriate for transplantation^13^.

In this study, we investigated newborn variables that may impact cord blood unit quality. This may help to establish a new standard for donor selection in our community as well as criteria for the general collection and preservation of UCB units.

## **Methods**

### Donor eligibility criteria

At the Obstetrics Department of Mansoura University Hospital in Egypt, mothers scheduled for elective cesarean section (CS) were approached upon admission for delivery. They were provided with a written information leaflet in Arabic detailing the donation process. Each mother was given 30 min to review the information before deciding whether to participate.

### Inclusion and exclusion criteria

The study included only women undergoing elective cesarean section deliveries. The exclusion criteria, based on hospital records, were as follows: Maternal age under 18 years or over 40 years (This decision was based on the increased risk of pregnancy-related complications, higher incidence of obstetric comorbidities, and potential alterations in cord blood characteristics in older mothers, which could impact the quality and usability of collected samples), fever > 38 °C, gestational age < 37 weeks, newborn weight < 2000, ruptured membranes ≥ 18 h before delivery, positive serologic tests for HBV, HCV, syphilis, or HIV, presence of obstetric complications, family history of genetic illnesses, and multiple gestation.

### Consent process

Potential donors who met the eligibility criteria were asked to complete a screening form with clinical questions. If eligible, they were invited to provide written informed consent. If eligible, they were informed about the potential use of cord blood for research purposes and its storage if it met the criteria for donation. The consent form was reviewed and signed before delivery, and a copy was filed in the hospital records.

The study was approved by the Mansoura Faculty of Medicine Institutional Research Board (R.21.12.1564.R1) and conducted following the guiding principles of the Declaration of Helsinki^14^. All methods were performed in accordance with the relevant guidelines Postpartum blood loss was closely monitored throughout the procedure, with no observed increase in hemorrhagic risk compared to standard cesarean section deliveries. This ensured the safety of the mothers while maintaining the integrity of the UCB collection process.

### Collection of umbilical cord blood units

In accordance with the methods of Bassiouny et al.^15^ and Korkor et al.^16^, UCB was collected in utero about (60 s/1 minute) after delivery of the fetus and before delivery of placenta acknowledging the established neonatal benefits, including improved hemoglobin levels, enhanced iron stores, and better circulatory stability. Utero tonics (e.g., oxytocin/carbetocin) were administered as per standard clinical protocols to facilitate placental separation. A povidone-iodine swab and a 70% alcohol solution were used to clean the umbilical cord. The umbilical vein at the distal end of the cord was punctured, and the cord blood collection bag was fitted with a large-calibre needle, allowing the blood to flow downwards until it stopped.

### Umbilical cord blood unit examination for bacterial contamination

The automated BACT/ALERT^®^ 3D Microbial Detection System (Biomeriux, Chemin dew l′Orme, France) was used to investigate UCB for bacterial contamination. This system is based on colorimetric detection, which changes color as pH rises and as a result of the rising carbon dioxide produced by the growing bacteria. After the membrane was cleaned, 10 mL of UCB samples were transferred into BACT/ALERT^®^ aerobic and anaerobic culture flasks (BACT/ALERT FA/FN). The cultures were stored for a total of ten days. Subcultures on agar plates were used to validate microbial growth when positive culture bottles were unloaded from the BACT/ALERT system. Any contaminated samples were thrown away and excluded from the research.

### Umbilical cord blood unit assessment

Upon collection, the UCB was preserved in a refrigerator at 4 °C until processing. It was then transferred to the UCB processing laboratory at Mansoura University Children’s Hospital and placed at a temperature of 4–24 °C in an isothermal bag. Their identification, physical integrity, and appearance were checked. Assuming that one gram of cord blood is equivalent to 0.95 milliliters, the weight of the dry unit (42 g) and the volume of anticoagulant (30 ml) were subtracted from the total volume to determine the final volume of the UCB.

### Cell enumeration

#### TNC determination

The enumeration tests were conducted in the hematology laboratory of Mansoura University’s Oncology Center. An automated cell counter (CELL-DYN 3700; Abbott, USA) was used to analyse TNC.

#### CD34 + cell count determination

CD34 + cells were counted via flow cytometry (EPICS XL; Beckman Coulter, USA) and the International Society for Hematotherapy and Graft Engineering (ISHAGE) protocol^17^. Monoclonal antibodies against CD34 + and CD45 + cells conjugated to fluorescein and phycoerythrin (Stem-Kit Reagents, Beckman Coulter, USA), respectively, were used to incubate UCB. 7-Aminoactinomycin D (7-AAD) dye was used to differentiate between dead and living cells. Beckman-Coulter XL Flow Cytometer - System II Software Version 3.0 (https://www.beckman.com/flow-cytometry/software*)* was used to analyse the data.

### Statistical analysis

Descriptive statistics were employed for cord blood units. Student’s t test was used to determine the differences between the two groups. The correlation between the variables was determined via Pearson’s correlation coefficient (r). *P* values less than 0.05 were regarded as significant. The Statistical Package for Social Sciences (SPSS 26) was used to process the data. Linear regression analysis via a stepwise forward approach was used to assess the independent factors affecting the outcomes.

## Results

In utero, 150 cord blood units in total were obtained. The collected cord blood had a mean volume of 88.51 ± 39.1 ml, with a range of 28.6–199.3 ml. The average number of TNCs acquired per UCB unit collected was 6.40 + 3.07 × 10^8^, as determined by the comparison of the volume of UCB versus the number of TNCs. The mean CD34 + cell count was 2.77 ± 1.81 × 10^6^ per UCB unit, ranging from 0.36 to 9.04 × 10^6^ per UCB unit (Table 1).

**Table 1:** Descriptive data for the 150 collected UCB units.

UCB data	*N*	Mean + SD	Median	Range
Cord blood volume (ml)	150	88.51 + 39.1	82	28.6–199.3
TNC (x108/UCB unit)	150	6.40 + 3.07	5.78	1.02–17.57
CD34 + cell (x106/UCB unit)	150	2.77 + 1.81	2.48	0.36–9.04

Units obtained from male neonates had a substantially larger cord blood volume than units obtained from female neonates (*P* = .029). However, there was an insignificant correlation between neonatal sex and either the TNC count (*P* = .615) or the number of CD34 + cells (*P* = .329). But, there was a significant difference regarding cord blood volume between male and females (*P* = .029) as it was (95.46 + 46.56) in male and (81.57 + 28.54) in female. The characteristics of the neonate, placenta, and umbilical cord are summarized in Table 2.

**Table 2:** Characteristics of the neonate, placenta, and umbilical cord.

Variable	Unit/Category	*N* (%)	Mean + SD	Median	Range
Sex	Male	75 (50%)			
	Female	75 (50%)			
Birth order (Primigravida/Multigravida)*	1st	20 (13.3%)			
	2nd	41 (27.3%)			
	3rd	69 (46%)			
	4th	12 (8%)			
	5th	8 (5.3%)			
Gestational age	Weeks	150	38.30 + 1.05	38	37–40
Birth weight	Grams	150	3491 + 421	3550	2550–4100
Head circumference	Cm	150	34.84 + 1.51	35	31–38
Chest circumference	Cm	150	33.16 + 3.02	33.35	28.5–36.0
Apgar score (1 min)	3	2 (1.3%)			
	4	6 (4%)			
	5	1 (0.7%)			
	6	27 (18%)			
	7	72 (48%)			
	8	27 (18%)			
	9	15 (10%)			
Apgar score (5 min)	7	1 (0.7%)			
	8	3 (2%)			
	9	6 (4%)			
	10	140 (93.3%)			
Placental weight	Grams	150	619 + 139	600	340–920
Cord length	Cm	150	58.68 + 1.29	58.5	34–90

* First birth order refers to the primgravida while from 2nd to 5th indicates multigravida.

**Fig. 1:**
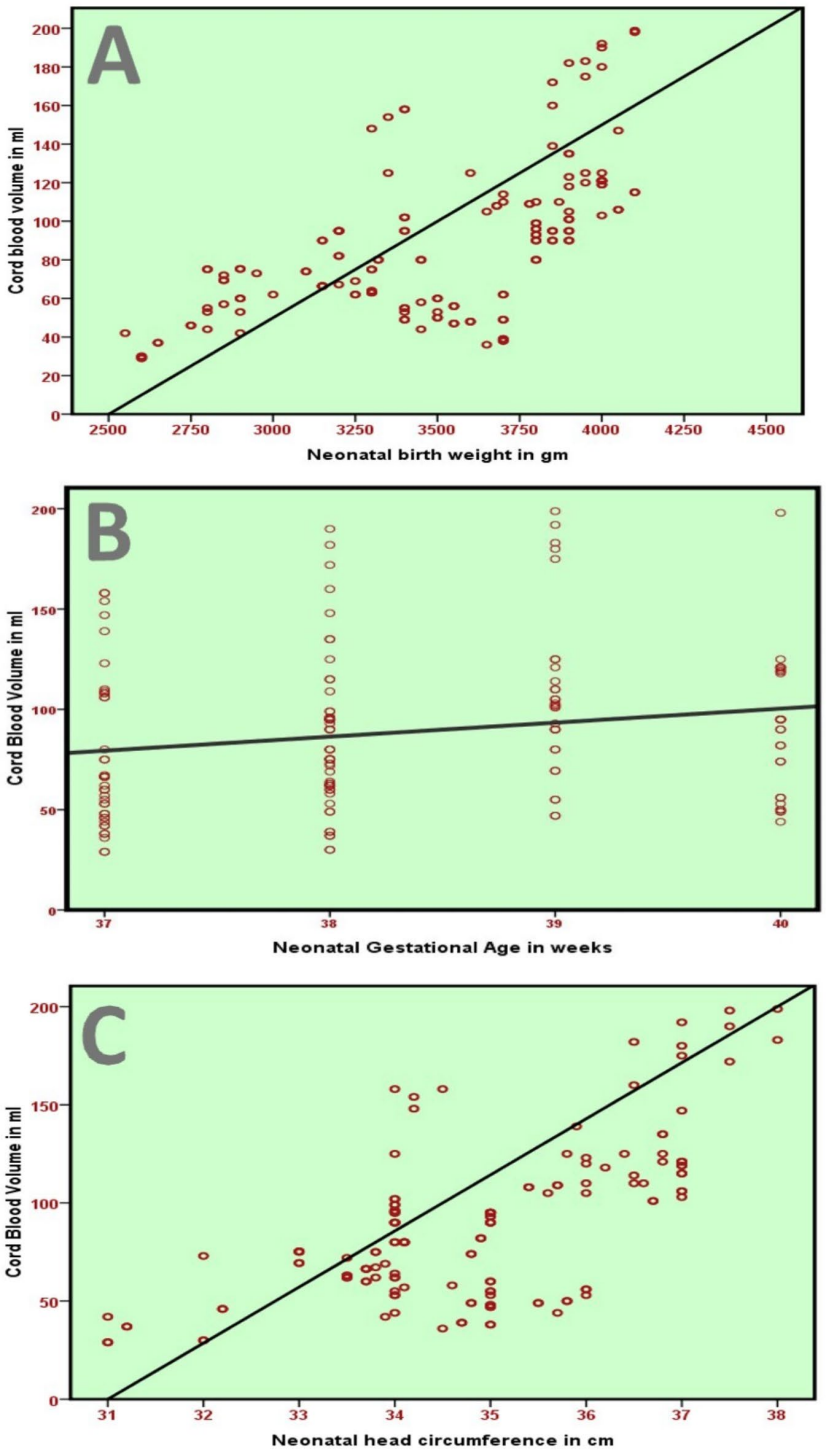
Scatter plot correlating the cord blood volume with (**A**) neonatal birth weight, (**B**) gestational age at birth, and (**C**) neonatal head circumference.

The birth weight, gestational age at birth, and head circumference (Fig. 1), and chest circumference of the newborn were significantly correlated with the volume of cord blood. A highly significant correlation was also found between both placental weight and cord length and cord blood volume (Table 3).

**Table 3:** Summary of univariate analysis for correlations.

	*N* (150)	Cord blood volume (ml)		TNC (x10^6^/ml)		CD34 + cell (x10^3^/ml)	
		Pearson correlation (r)	p value	Pearson correlation (r)	p value	Pearson correlation (r)	p value
Birth order	150	0.152	0.064	0.138	0.092	0.218	0.007
Gestational age	150	0.187	0.022	0.080	0.328	0.327	< 0.001
Birth weight	150	0.616	< 0.001	0.306	< 0.001	0.755	< 0.001
Head circumference	150	0.661	< 0.001	0.755	< 0.001	0.673	< 0.001
Chest circumference	150	0.483	< 0.001	0.379	< 0.001	0.673	< 0.001
Apgar score (1 min)	150	0.033	0.686	0.092	0.265	0.120	0.144
Apgar score (5 min)	150	-0.151	0.066	0.471	0.081	-0.145	0.076
Placental weight	150	0.557	< 0.001	0.381	< 0.001	0.610	< 0.001
Cord length	150	0.531	< 0.001	0.437	< 0.001	0.514	< 0.001

There was a highly significant correlation between the TNC count/ml and the ratio of the CD34 + cell count/ml to the birth weight, head circumference, and chest circumference of the newborn and both the placental weight and cord length.

The birth order and the CD34 + cell count/ml showed a highly significant correlation. There was no correlation between the cord blood volume, CD34 + cell count/ml, TNC count/ml, and Apgar score (at 1 min and 5 min). A multivariate linear regression analysis was employed to establish the primary selection criteria of the UCB units. The birth order, chest circumference, head circumference, cord length, Apgar score (1 min), and Apgar score (5 min) are not considered predictive variables for stem cell yield from UCB units.

Our findings suggest that optimal UCB collection is influenced by several neonatal and placental factors. Key determinants include higher birth weight (> 3.0 kg), greater placental weight (≥ 600 g), and longer umbilical cord length (≥ 58 cm), all of which significantly correlate with increased UCB volume, TNC count, and CD34 + cell yield. As shown in Table 4, placental weight was the key predictive factor for all umbilical cord blood (UCB) unit parameters. Additionally, neonatal birth weight was specifically predictive of UCB volume and CD34 + cell count. These findings underscore the importance of fetal and placental growth in optimizing UCB collection for stem cell banking.

**Table 4:** Multivariate analysis findings of neonatal variables influencing UCB units.

Outcome indicator	Predictor variable	Standardized coefficients beta	*P* value	95% CI
CBU Volume	Neonatal birth weight in gm	0.457	< 0.001	0.024-0.061
	Placental weight in gm	0.580	< 0.001	0.033-0.075
TNC	Placental weight in gm	0.381	< 0.001	0.002-0.006
CD34+	Neonatal birth weight in gm	0.521	< 0.001	0.006-0.013
	Gestational Age in weeks	0.216	< 0.001	0.699-2.427
	Placental weight in gm	0.235	0.001	0.003-0.022

## Discussion

Because umbilical cord blood is rich in hematological stem cells (HSCs) that can be reconstituted after storage, it is a source of stem cells used to manage hematological disorders^18^. Research has indicated that the number of TNCs transplanted determines the engraftment of cord blood stem cells, and the majority of cord blood units do not contain sufficient stem cells for adults to successfully reconstitute their bone marrow^19^. As a consequence, determining the best parameters for collecting cord blood and evaluating the quality of UCB before preservation is crucial. Many previous studies have correlated neonatal factors with the quality of cord blood units. However, only limited information is available in Egypt. The current study aimed to assess whether neonatal variables influence the quality of the cord blood units that were collected.

In the present study, UCB was obtained before placental delivery. The collection from the placenta in utero is simple and does not interfere with a baby’s natural delivery process. It is a standardized collection technique that ensures optimal sample volume and reduces contamination risk. UCB was obtained during Cesarean section deliveries according to Yamada et al.^20^ and Bassiouny et al.^15^, and the UCB volume was approximately greater than that obtained during vaginal deliveries. A study by Mancinelli et al.^21^ offers a possible explanation for the greater volume of cord blood collected during Cesarean section. They argued that the influence of gravity, as the infant is placed above the placenta before cord clamping, may play a role in promoting the downflow of blood into the placental cord and, ultimately, into the placental compartment. A further rationale is that the placenta is physically removed from the human body more quickly following a Cesarean surgery than it occurs after a vaginal birth, which reduces the risk of blood clot formation^15^.

The mean cord blood volume collected and the TNC and CD34 + cell counts (88.51 + 39.1 ml, 6.40 + 3.07 × 10^8^ and 2.77 + 1.81 × 10^6^) in the present study were consistent with those reported in other studies worldwide^22,23^. According to the present investigation, there was a significant correlation between cord blood volume and male newborns. Additional research revealed no correlation between the volume of cord blood obtained and the sex of the newborn^24^. Nevertheless, there was no significant correlation between newborn sex and the TNC count/ml. Furthermore, no significant correlation was found between newborn sex and CD34 + cells.

Conversely, studies have demonstrated that variables such as sex could impact the number of CD34 + cells^24^.

As expected, there was a significant correlation between longer cord length and larger placenta and cord blood volumes, TNC counts, and CD34 + cell concentrations. Furthermore, units taken from neonates with higher birth weights than from those with lower birth weights presented greater blood volume, a higher CD34 + cell count, and a higher TNC count, which is consistent with the findings previously described^21,25–27^. This might be explained by the fact that babies born when weighing more have better nutritional conditions, which results in increased placental volumes and higher volumes of UCB.

Interestingly, higher birth order and longer gestational age were correlated with greater CD34 + cell counts. Regarding gestational age, Mancinelli et al.^21^ reported that a gestational age of more than 39 weeks considerably increased the number of CD34 + patients. This may be explained by the placental aging that occurs with increasing gestational age, which in turn can lead to progressive fetal hypoxia, resulting in an increase in hematopoietic stem cells and cord blood volume.

Ballen et al.^25^ demonstrated that cord units with greater TNC levels were produced by women with fewer previous live births, which contrasts with our results on birth order. The reason for this discrepancy might be attributed to the larger number of babies in the second/third delivery in our research, which may have countered the impact of birth order.

The cord blood volume, TNC yield, and CD34 + cell concentration were significantly positively correlated with neonatal head circumference and chest circumference. To the best of our knowledge, this is the first study to investigate the effects of these two neonatal factors on cord blood unit quality. Surprisingly, the cord blood volume and CD34 + and TNC cell counts were not correlated with the Apgar score. This finding was inconsistent with previous data^28^.

A multivariate linear regression analysis was carried out to establish the primary selection criteria of the UCB units. Placental weight is assumed to be a good predictive variable for selecting UCB units, as a heavier placenta is positively correlated with greater volume and increased counts of CD34 + and TNC cells. This finding is in agreement with that of Solves et al.^29^ and García et al.^30^, who recommended that placental weight estimation could be included in the criteria for cord blood donors to increase bank efficiency. Additionally, Marzouk and Abd-Allah^31^ concluded that a placental weight ≥ 550 g is considered a predictive variable for stem cell yield.

In conclusion, our research demonstrated that larger newborns, longer cords, larger placentas, and greater gestational ages produce higher yields of stem cells. The cell yield of the cord blood unit is unaffected by neonatal sex. The placental weight and newborn birth weight are considered key predictive indicators impacting the quantity and quality of UCB units and their banking efficiency.

We acknowledge the challenges posed by the inability of newborns to give consent for such procedures. In line with ethical guidelines, cord blood collection is typically carried out under the assumption that the mother provides informed consent on behalf of the newborn, as the procedure is often considered a minimal risk to the infant and provides potential benefits for medical research or stem cell banking^32^ and in accordance with established ethical guidelines, parental consent serves as the standard framework for neonatal medical decisions, including surgical interventions and research participation. Since the placenta and umbilical cord are considered biological extensions of the maternal-fetal unit, their use under maternal consent is ethically justified. Prior to collection, all prospective donors were thoroughly informed about the purpose, risks, and potential benefits of UCB donation, ensuring voluntary and informed decision-making. Additionally, the study adhered to stringent confidentiality and data protection measures, safeguarding donor identities and medical information. In many countries, such as those following the Declaration of Helsinki, guidelines^14^ for obtaining consent are well-established and require clear communication with the mother about the intended use of the collected cord blood, along with respect for her autonomy and the newborn’s welfare. Regulatory bodies ensure that the collection is done ethically and that the rights of both the mother and the child are safeguarded.

## Data Availability

All data generated or analyzed during this study are included in this published article.

## References

[1] Tu, M. et al. A predictive model combining clinical characteristics and nutritional risk factors for overall survival after umbilical cord blood transplantation. *Stem Cell. Res. Ther.* **14** (1), 304 (2023). 37872622 10.1186/s13287-023-03538-7 PMC10594692

[2] Zhu, X., Tang, B. & Sun, Z. Umbilical cord blood transplantation: still growing and improving. *Stem Cells Transl. Med.* **10** (S2), S62–74 (2021). 34724722 10.1002/sctm.20-0495 PMC8560197

[3] Sanchez-Petitto, G. et al. Umbilical cord blood transplantation: connecting its origin to its future. *Stem Cells Transl. Med.* **12** (2), 55–71 (2023). 36779789 10.1093/stcltm/szac086 PMC9985112

[4] Laue, J., Ambühl, J. & Surbek, D. Hybrid umbilical cord blood banking: literature review. *Arch. Gynecol. Obstet.* **309** (1), 93–104 (2024). 37093267 10.1007/s00404-023-07003-x PMC10124678

[5] Navarrete, C. & Contreras, M. Cord blood banking: a historical perspective. *Br. J. Haematol.* **147** (2), 236–245 (2009). 19796273 10.1111/j.1365-2141.2009.07827.x

[6] McCullough, J., McKenna, D., Kadidlo, D., Schierman, T. & Wagner, J. Issues in the quality of umbilical cord blood stem cells for transplantation. *Transfusion (Paris)* **45** (6), 832–841 (2005). 10.1111/j.1537-2995.2005.04265.x 15934980

[7] Wagner, J. E. et al. Transplantation of unrelated donor umbilical cord blood in 102 patients with malignant and nonmalignant diseases: influence of CD34 cell dose and HLA disparity on treatment-related mortality and survival. *Blood J. Am. Soc. Hematol.* **100** (5), 1611–1618 (2002). 10.1182/blood-2002-01-0294 12176879

[8] Styczynski, J. et al. Outcomes of unrelated cord blood transplantation in pediatric recipients. *Bone Marrow Transpl.* **34** (2), 129–136 (2004). 10.1038/sj.bmt.1704537 15107815

[9] Novelo-Garza, B. et al. Establishing a cord blood banking and transplantation program in Mexico: a single institution experience. *Transfusion (Paris)* **48** (2), 228–236 (2008). 10.1111/j.1537-2995.2007.01529.x 18028272

[10] Lown, R. et al. Ethnicity, length of time on the register and sex predict donor availability at the confirmatory typing stage. *Bone Marrow Transpl.* **49** (4), 525–531 (2014). 10.1038/bmt.2013.206 24419516

[11] Ballen, K. K., Barker, J. N., Stewart, S. K., Greene, M. F. & Lane, T. A. Collection and preservation of cord blood for personal use. *Biol. Blood Marrow Transpl.* **14** (3), 356–363 (2008). 10.1016/j.bbmt.2007.11.005 18275904

[12] Welte, K., Foeken, L., Gluckman, E. & Navarrete, C. International exchange of cord blood units: the registry aspects. *Bone Marrow Transpl.* **45** (5), 825–831 (2010). 10.1038/bmt.2010.11 20190837

[13] Hollands, P. & McCauley, C. Private cord blood banking: current use and clinical future. *Stem Cell. Rev. Rep.* **5**, 195–203 (2009). 19603288 10.1007/s12015-009-9082-0

[14] Ashcroft, R. E. The declaration of Helsinki. *Oxf. Textb. Clin. Res. Ethics* 141–148. (2008).

[15] Bassiouny, M., El-Chennawi, F., Mansour, A., Yahia, S. & Darwish, A. Optimal method for collection of umbilical cord blood: an E Gyptian trial for a public cord blood bank. *Transfusion (Paris)* **55** (6), 1263–1268 (2015). 10.1111/trf.12978 25565448

[16] Korkor, M. S., Khashaba, M., Mohamed, S. A. & Darwish, A. Effect of different timings of umbilical cord clamping on the level of CD34 + cells in full-term neonates. *Sci. Rep.* **13** (1), 22917 (2023). 38129640 10.1038/s41598-023-50100-9 PMC10739938

[17] Sutherland, D. R., Anderson, L., Keeney, M., Nayar, R. & Chin-Yee, I. The ISHAGE guidelines for CD34 + cell determination by flow cytometry. *J. Hematother.* **5** (3), 213–226 (1996). 8817388 10.1089/scd.1.1996.5.213

[18] Rocha, V., Gluckman, E. & Eurocord-Netcord registry and European blood and marrow transplant group. Improving outcomes of cord blood transplantation: HLA matching, cell dose and other graft‐and transplantation‐related factors. *Br. J. Haematol.* **147** (2), 262–274 (2009). 19796275 10.1111/j.1365-2141.2009.07883.x

[19] Kurtzberg, J. et al. Unrelated donor cord blood transplantation in children: lessons learned over 3 decades. *Stem Cells Transl. Med.* **12** (1), 26–38 (2023). 36718114 10.1093/stcltm/szac079 PMC9887081

[20] Yamada, T. et al. Factors affecting the volume of umbilical cord blood collections. *Acta Obstet. Gynecol. Scand. Orig. Artic.* **79** (10), 830–833 (2000). 11304964

[21] Mancinelli, F. et al. *Optimizing Umbilical Cord Blood Collection: Impact of Obstetric Factors Versus Quality of Cord Blood Units* 1174–1176 (Elsevier, 2006). 10.1016/j.transproceed.2006.03.052 16757298

[22] Ademokun, J. et al. Umbilical cord blood collection and separation for Haematopoietic progenitor cell banking. *Bone Marrow Transpl.* **19** (10), 1023–1028 (1997). 10.1038/sj.bmt.1700788 9169647

[23] Armitage, S. et al. Cord blood banking: volume reduction of cord blood units using a semi-automated closed system. *Bone Marrow Transpl.* **23** (5), 505–509 (1999). 10.1038/sj.bmt.1701591 10100566

[24] Nakagawa, R. et al. Analysis of maternal and neonatal factors that influence the nucleated and CD34 + cell yield for cord blood banking. *Transfusion (Paris)* **44** (2), 262–267 (2004). 10.1111/j.1537-2995.2004.00645.x 14962318

[25] Ballen, K. et al. Bigger is better: maternal and neonatal predictors of hematopoietic potential of umbilical cord blood units. *Bone Marrow Transpl.* **27** (1), 7–14 (2001). 10.1038/sj.bmt.1702729 11244432

[26] Nunes, R. D. & Zandavalli, F. M. Association between maternal and fetal factors and quality of cord blood as a source of stem cells. *Rev. Bras. Hematol. E Hemoter.* **37** (1), 38–42 (2015). 10.1016/j.bjhh.2014.07.023 PMC4318845 25638766

[27] Prakash, S., Jain, A., Pahwa, D., Kalra, J. K. & Sharma, R. Maternal and neonatal variables affecting CD34 + cell count in the umbilical cord blood. *J. Appl. Hematol.* **13** (1), 41–46 (2022).

[28] Elchennawy, A., Mohammed, F. Z., Elghazaly, F. A. & Eldesouky, A. F. Factors affecting hematopoietic stem cells derived from umbilical cord blood. *Biochem. Lett.* **15** (1), 126–146 (2019).

[29] Solves, P. et al. Maternal, neonatal and collection factors influencing the Haematopoietic content of cord blood units. *Acta Haematol.* **113** (4), 241–246 (2005). 15983430 10.1159/000084677

[30] García, S. Donors selection and retrieval of units in an umbilical cord blood bank. *Med. Clin. (Barc.)* **129** (15), 561–565 (2007). 17988611 10.1157/13111706

[31] Marzouk, T. & Abd-Allah, I. M. Maternal and neonatal predictive variables for quality of cord blood as a source of stem cells. *J. Nurs. Educ. Pract.* **8** (11). (2018).

[32] Bień, A., Vermeulen, J., Bączek, G., Pięta, M. & Pięta, B. Cord blood banking: balancing hype and hope in stem cell therapy. *Eur. J. Midwifery* **8**, 10–8332 (2024). 10.18332/ejm/192930 PMC11456975 39376486

